# Optimization of dry period in Karan Fries cow

**DOI:** 10.14202/vetworld.2016.648-652

**Published:** 2016-06-24

**Authors:** K. Puhle Japheth, R. K. Mehla, Mahendra Singh, A. K. Gupta, Ramendra Das, Pranay Bharti, T. Chandrasekar

**Affiliations:** 1 Division of Livestock Production Management, ICAR-National Dairy Research Institute, Karnal - 132 001, Haryana, India; 2 Division of Dairy Cattle Physiology, ICAR-National Dairy Research Institute, Karnal - 132 001, Haryana, India; 3 Division of Dairy Cattle Breeding, ICAR-National Dairy Research Institute, Karnal - 132 001, Haryana, India

**Keywords:** dry period, economic trait, Karan Fries cow, non-genetic factors, optimization

## Abstract

**Aim:**

The objective of this study was to optimize dry period (DP) length that can maximize the production across adjacent lactations and overall lifetime yield.

**Materials and Methods:**

Performance records with respect to DP spread over a period of 15-year in Karan Fries (KF) cattle maintained at Livestock Research Centre (National Dairy Research Institute), were collected for the study. Data of 681 KF cows were analyzed by least square technique to examine the effect of non-genetic factors on DP. Season of calving was classified into four seasons: Winter season (December-March), summer season (April-June), rainy season (July-September), and autumn season (October-November); period of calving into five periods: 1998-2000 (1-period), 2001-2003 (II-period), 2004-2006 (III-period), 2007-2009 (IV-period), and 2010-2012 (V-period), and parity into six parities, i.e., 1^st^, 2^nd^, 3^rd^, 4^th^, 5^th^, and >6^th^ parities to see the effect of non-genetic factors on DP.

**Results:**

Period of calving, season of calving, and parity did not affect the DP significantly (p<0.05). The overall least square mean of DP was 67.93±2.12 days. For the optimization of DP with regard to milk productivity, analysis was carried out by class interval method. DP was classified into eight classes (<22, 23-45, 46-67, 68-89, 90-111, 112-133, 134-155, and >156 days), and optimum level was obtained at 46-67 days (3^rd^ class) with the following respective milk yield (MY) of 305 daily MY (4016.44±43.68 kg), total MY (4704.21±61.51 kg), MY per day of lactation length (13.03±0.13 kg), and MY per day of calving interval (11.68±0.41 kg).

**Conclusion:**

From the study, it was concluded that this optimal DP length (46-67 days) is suitable for maximizing the production. Hence, one should aim to dry off pregnant cows to achieve a DP of appropriate length to enhance productivity in the next lactation, as very short and very long DP reduces the economic profitability in dairy animals.

## Introduction

The economic survey 2011 analyzed the dairy situation in India, considering that the requirement of milk in 2021-2022 is expected to be 180 million tons as against the current level of milk production of 127.3 million tons [1]. Dairy managers aim to dry off pregnant cows to achieve a dry period (DP) of appropriate length to maximize productivity in the next lactation [2]. A DP of 60 days is considered as ideal [3]. DP <30 days (in US Holstein cow) are detrimental to lifetime yield and should be avoided [4]. The fetus completes almost two-thirds of its growth during the last 2 months of gestation. This fetal growth takes priority over the cow’s own needs for body tissue maintenance. The rumen papilla and microflora must adapt to the change from an energy dense lactating ration to one that meets basic maintenance requirements, and then prepare again during the transition period to adjust back to the lactating ration.

So, this is a period of anatomical and physiological challenging time for the cow and her udder [5]. It is a time of nutritional, metabolic, and mammary change that will profoundly impact health and productivity in the next lactation. Another principle factor for causing the variation in milk yield (MY) and calving interval (CI) is DP thus, influencing the efficiency of MY in dairy cow. A reasonable length of DP is necessary because this period provides time to regain the energy lost during the previous lactation and to regenerate the secretory cells of animal for next lactation. Despite many remarkable performances, there is a wide range of variability in the range of DP of Karan Fries (KF) cattle that hampered the productive as well as reproductive performance of the cows.

Therefore, the study was conducted with the objective to find out the optimum level of DP in a narrower range which would be considered as optimum so as to improve milk production not only in the particular lactation but also in the overall performance of the animal by enhancing genetic gain.

## Materials and Methods

### Ethical approval

The present study was carried out after getting approval by the Research Committee and Institutional Animal Ethic Committee of National Dairy Research Institute.

### Experimental site

The study was conducted at the National Dairy Research Institute (NDRI), Karnal, India. The farm is located at an altitude of 245 m above the sea level, in the Indo-Gangetic alluvial plains at 29° 42’N and latitude 72° 54’ E longitude. The climate of this region is subtropical in nature with temperature ranging between 2°C in winter and 45°C in summer. The area receives an annual rainfall of 760-960 mm mostly during July and August with relative humidity ranging from 41% to 85%. Thus, it is obvious the cattle maintained at NDRI farm were exposed to extreme climate conditions due wide range of meteorological variation.

### Housing management

The KF cattle in the farm were kept under loose housing system. The open paddocks are brick on edge flooring system with large space available to provide adequate exercises. The pregnant cows were transferred to the maternal pen 2 weeks before the actual date of calving. Pregnant cows were provided single pen with ample space 12 m^2^ × 12 m^2^ for covered and opened area (Bureau of Indian Standards standard), proper ventilation, and drainage system. During summer season, cows were provided with provision of fan and water sprinklers to mitigate heat stress.

### Feeding and other management

The nutritional requirement of KF cattle was met through both roughages and concentrate. The farm practices *ad libitum* feeding thrice a day (i.e. morning, afternoon, and evening) with good quality green fodder throughout the year such as berseem, lucern, cowpea, maize, jowar, bajra, and wheat. Silage and hay were also used during the lean season. Concentrate was provided to the cows as per their milk production at the time of milking (i.e. morning, afternoon, and evening). The cows accessed to *ad libitum* fresh drinking water day and night.

Both hand milking and machine milking were practice in the farm. Milking is done thrice a day, i.e. early morning, afternoon, and evening. The average MY in a lactation and MY per day were 4677.84±50.35 kg and 12.93±0.99 kg, respectively [6]. All the sanitary care and measure were taken before, during, and after milking. All types of veterinary aids, prophylactic, and sanitary measures were taken care.

### Collection of data

The data of production traits of 681 KF cow were collected and utilized for the study from the period of 1998 to 2012 (15 years). The record of cows with abnormal calvings such as premature calving, abortion, and shorter lactation length (LL) was omitted in the study. The lactation records of 250 days and above were considered as normal and included in the study to see the effects of season of calving, period of calving, and parity on the DP. The data were classified and coded according to different seasons: Winter season (December-March), summer season (April-June), rainy season (July-September), and autumn season (October-November). For period of calving, it was classified into five periods: 1998-2000 (I-period), 2001-2003 (II-period), 2004-2006 (III-period), 2007-2009 (IV-period), and 2010-2012 (V-period). Finally for parities, the data are classified into six classes: 1^st^, 2^nd^, 3^rd^, 4^th^, 5^th^, and <6^th^ (parities six and above were combined due to lesser animal number of observation) to observe the effect of non-genetic factors on DP.

### Statistical analysis

The data were subjected to least-squares technique [7] to see the effects of season of calving, period of calving, and parity on DP. Duncan’s multiple range test was used to test the significance of differences between treatments’ means. The least squares analysis model is given as:

Y_ijkl_ = μ + S_i_ + P_j_ + A_k_ + e_ijkl_

Where,

Y_ijkl_= Dependent trait (DP) of I^th^ cow born in i^th^ season, j^th^ period, and k^th^ parity,

μ=Overall mean,

S_i_=Effect of i^th^ season of calving (i=1-4),

P_j_=Effect of j^th^ period of calving (j=1-5),

A_k_=Effect of k^th^ parity (k=1-6),

e_ijkl_= Random error, NID with zero and constant variance (0,σ_e_^2^).

### Optimization of DP

For optimization of DP with regard to milk productivity, the various DP lengths were classified into eight (1-8) classes (Table-1). Class interval for DP was calculated with the help of Sturges formula.

**Table-1a:** Average 305 DMY and TMY (kg) for different classes of DP in KF cow.

Class of DP (days)	Number of observation	Percentage of animals	Cumulative (%)	Average 305 DMY (kg)	Average TMY (kg)
<22	28	2.86	2.86	4037.0±201.55	4705.34±283.82
23-45	84	8.58	11.44	4050.25±116.36	4686.48±163.86
46-67	596	60.94	72.38	4016.44±43.68	4704.21±61.51
68-89	114	11.65	84.03	3879.8±99.89	4497.0±140.66
90-111	72	7.36	91.39	3833.98±125.69	4391.97±176.99
112-133	30	3.06	94.45	3939.77±194.72	4788.77±274.19
134-155	26	2.65	97.10	4171.07±209.16	5268.21±294.53
>156	28	2.86	100	3489.79±201.55	4319.0±283.82

DP=Dry period, KF=Karan Fries, TMY=Total milk yield, DMY=Daily milk yield

**Table-1b:** Average MY/LL and MY/CI (kg) for different classes of DP in KF cow.

Class of DP (in days)	Number of observation	Percentage of animals	Cumulative (%)	MY/LL (kg)*	MY/CI (kg)*
<22	28	2.86	2.86	11.79±0.63^abc^	12.93±1.93^b^
23-45	84	8.58	11.44	12.98±0.36^bc^	11.72±1.11^ab^
46-67	596	60.94	72.38	13.03±0.13^c^	11.68±0.41^b^
68-89	114	11.65	84.03	12.12±0.31^ab^	10.02±0.95^ab^
90-111	72	7.36	91.39	11.86±0.39^a^	9.30±1.20^ab^
112-133	30	3.06	94.45	11.82±0.61^abc^	8.99±1.86^ab^
134-155	26	2.65	97.10	12.23±0.65^abc^	9.09±2.00^ab^
>156	28	2.86	100	10.83±0.63^a^	7.45±1.93^a^

* Significant at 5% level (p<0.05) and the values with different superscripts within a column differ significantly. DP=Dry period, KF=Karan Fries, MY/LL=Milk yield per day of lactation length, MY/CI=Milk yield per day of calving interval

C = R/1 + 3.322 log 10N

Where,

C=Width of class/class interval,

N=Number of observations,

R=Range (maximum-minimum),

1+3.322 log10N=Number of classes.

The average MYof different classes of the DP was studied using least squares analysis. The model used is given below:

Y_ij_ = μ + C_i_ + e_ij_

Where,

Y_ij_ = j^th^ observation of i^th^ class of DP,

C_i_ = Effect of i^th^ class of DP,

I = 1, 2., 8 class of DP,

e_ij_ = Random error, assumed to be normally and independently distributed with mean zero and constant variance, i.e. NID (0, σe2).

## Results

### Effect of non-genetic factors on DP

In the study, the overall least squares means of DP was 67.93±2.12 days (Table-2). It was observed that season of calving, period of calving, and parity had no statistically significant effect on the DP. The longest DP was observed in those cows which calved during autumn season (70.03±2.67 days), whereas, the shortest DP was observed in rainy season (66.42±2.38 days). For the period of calving, the longest DP was observed in the III-period (71.32±2.11 days), whereas shortest average mean of DP was observed in I-period (62.46±2.96 days). In case of parity, there is no much variation in the average mean DP length across the different lactations.

**Table-2:** Least square means±SE value and effects of nongenetic factors on DP in KF cow.

Parameters	Number of observation	Average means of dry period (days)
Overall means	965	67.93±2.12
Season of calving		
Winter season	356	67.46±1.99
Summer season	250	67.80±2.18
Rainy season	205	66.42±2.38
Autumn season	154	70.03±2.67
Period of calving		
I-period	136	62.46±2.96
II-period	207	68.03±2.38
III-period	240	71.32±2.11
IV-period	272	67.63±2.11
Vperiod	110	70.20±3.08
Parity		
1^st^ lactation	405	65.31±1.61
2^nd^ lactation	247	69.93±1.97
3^rd^ lactation	147	65.74±2.55
4^th^ lactation	83	69.11±3.40
5^th^ lactation	46	69.10±4.58
6^th^ and above lactation	37	68.37±5.07

SE=Standard error, DP=Dry period, KF=Karan Fries

### Optimization of DP

DP was divided into eight different classes by the use class interval method (Table-1a and b). The last two classes (i.e., 8^th^ and 9^th^ class) had been combined due to lesser numbers of animal observations. The average means of 305 days or less MY (305 daily MY [DMY]), total MY (TMY), MY per day of LL (MY/LL), and MY per day of CI (MY/CI) for each class of DP were estimated and presented (Table-1a and b).

### DP and 305 days or less MY and TMY

The estimated averages of 305 days or less MY and TMY for each class of DP were presented (Table-1a). The maximum 305 DMY (4171.07±209.16 kg) and TMY (5268.21±294.53 kg) were observed in the 7^th^ class (134-155 days). More than majority of the number of animal observations (60.94%) was observed in 3^rd^ class (46-67 days), while minimum numbers of animal observations are falls in 1^st^ and 8^th^ class, respectively with the same number of animal observations (2.86%) each. There is no significant effect of DP on 305 days or less MY and TMY.

### DP and MY/LL

The average MY/LL for each class of DP was estimated and presented (Table-1b). The animal group in the 3^rd^ class (46-67 days) showed the highest MY/LL (13.03±0.13 kg), whereas those animals in the 8^th^ class (134-155 days) revealed the least MY/LL (10.83±0.63 kg). Maximum number of animal observation (60.94%) was observed in 3^rd^ class (46-67 days), whereas minimum number of animal observation (2.86%) was found in 1^st^ and 8^th^ class, respectively (<22 and <156 days).

### DP and MY/CI

The averages of MY/CI for each class of DP were estimated and presented (Table-1b). The maximum MY/CI (11.72±1.11 kg) was observed in 2^nd^ class (23-45 days), whereas minimum MY/CI (8.99±1.86 kg) was found in 6^th^ class (112-133 days). The majority number of animal observations (60.94%) was in 3^rd^ class group (46-67 days), whereas the minimum number of animals (2.86%) observation was fall in 1^st^ class and 8^th^ class group (<22 and <156 days). There was a significant effect of DP on MY/LL and MY/CI.

## Discussion

From the study, the overall least squares means of DP was 67.93±2.12 days (Table-2), which was in agreement with Singh and Tomar [8], Singh [9], who also reported similar observation in KF cattle. Higher average DP was also reported by Nayak and Raheja [10], Singh *et al*. [11] in various HF cross with Zebu cows. It was observed that season of calving, period of calving, and parity had no significant effect on the DP. Similar results are also reported by Singh and Tomar [8], Singh [9] in HF crossbred. However, significant effects of the period of calving are reported by Thalkari *et al*. [12] and Javed *et al*. [13] in HF crossbred and indigenous cows. Furthermore, researchers such as Javed *et al*. [13,14] reported a significant effect of season of calving on DP in HF crossed with indigenous cows. The non-significant effect of parity on DP was reported by Rehman *et al*. [15] in indigenous cattle and Gatchearle *et al*. [16] in HF crossed with Deoni cattle. On the other hand, significant effects of parity on DP are reported in Friesian crossbred [17].

The rate of change of milk production in relations to change in length of DP shows that there was increase in milk production with increase in DP length till 3^rd^ class and then shows a slight decrease in the subsequent classes. Season of calving and lactation number did not influence on 305 DMY, TMY, MY/LL, and MY/CI as reported by Nehra and Divya *et al*. [18,19] while there was report of the significant effect of period of calving on 305 DMY in KF cattle [20]. However, various important factors (i.e., calving year, calving season, age groups, and parity) affect not only TMY but also the rate of milk production throughout the length of lactation [21]. The average mean of DP length for different parities was almost more or less the same.

## Conclusion

From the study, it was concluded that the production performance was better in the animal group which falls under the 3^rd^ class (46-67 days) with more than majority numbers of animal observations (60.94%). As in economical point of view, too short DP are not favorable, as it does not give proper rest to regain cows body condition for next lactation. However, very lengthy DP is also not profitable as it shortens the LL. Since DP is a crucial stage in the lactation cycle of a dairy cow, therefore, one should aim to dry off pregnant cows to achieve a DP of appropriate length to maximize productivity in the next lactation.

## Authors’ Contributions

KPJ, RKM, MS, and AKG designed the work. KPJ and RKM conducted the research work. Data analysis and manuscript were written by KPJ with the help of RD, PB, and TC under the guidance of RKM, MS, and AKG. All the authors have read and approved the final manuscript.
